# MYB exhibits racially disparate expression, clinicopathologic association, and predictive potential for biochemical recurrence in prostate cancer

**DOI:** 10.1016/j.isci.2023.108487

**Published:** 2023-11-19

**Authors:** Mohammad Aslam Khan, Srijan Acharya, Shashi Anand, Fnu Sameeta, Paramahansa Pramanik, Christopher Keel, Seema Singh, James Elliot Carter, Santanu Dasgupta, Ajay Pratap Singh

**Affiliations:** 1 Department of Pathology, College of Medicine, University of South Alabama, Mobile, AL 36617, USA; 2 Cancer Biology Program, Mitchell Cancer Institute, University of South Alabama, Mobile, AL 36604, USA; 3 Department of Mathematics and Statistics, University of South Alabama, Mobile, AL 36688, USA; 4 Department of Urology, Frederick P. Whiddon College of Medicine, University of South Alabama, Mobile, AL 36688, USA; 5 Department of Biochemistry and Molecular Biology, Frederick P. Whiddon College of Medicine, University of South Alabama, Mobile, AL 36688, USA

**Keywords:** Health disparity, Molecular biology, Cancer

## Abstract

MYB acts as a potentiator of aggressiveness and castration resistance in prostate cancer (PCa) through aberrant activation of androgen receptor (AR) signaling. Since Black men experience higher PCa incidence and mortality than White men, we examined if MYB was differentially expressed in prostate tumors from patients of these racial backgrounds. The data reveal that aberrant MYB expression starts early in precancerous high-grade prostate intraepithelial neoplastic lesions and increases progressively in malignant cells. PCa tissues from Black patients exhibit higher MYB expression than White patients in overall and grade-wise comparisons. MYB also exhibits a positive correlation with AR expression and both display higher expression in advanced tumor stages. Notably, we find that MYB is a better predictor of biochemical recurrence than AR, pre-treatment PSA, or Gleason’s grades. These findings establish MYB as a promising molecular target in PCa that could be used for improved risk prediction and therapeutic planning.

## Highlights

• MYB is aberrantly expressed in prostate cancer
• MYB expression increases progressively correlating with the advancing tumor grades
• A higher MYB expression is detected in Black patients than in White patients
• MYB is a promising predictive biomarker of biochemical recurrence

## Graphical abstract

**Figure:**
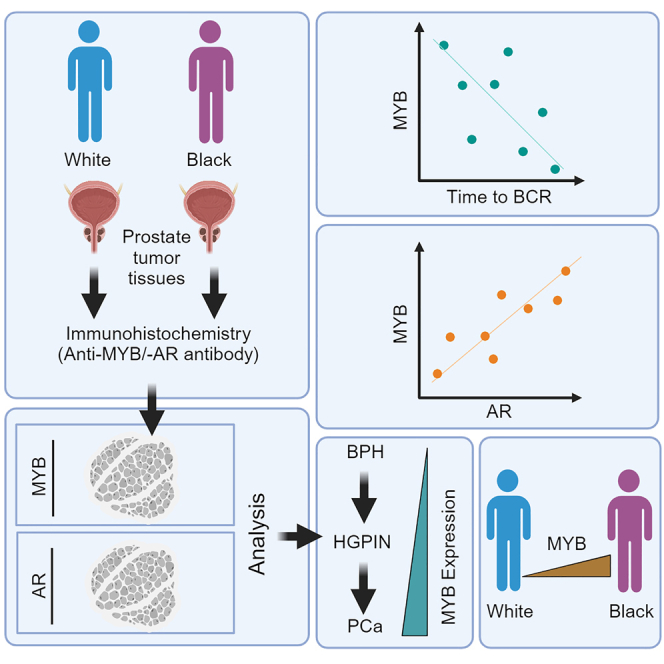

## Introduction

Prostate cancer (PCa) is the most common malignancy of the urinary tract and the second leading cause of cancer-related death in American men.^1^ It also has the widest racial health disparities among all cancers, which are apparent at every stage of the cancer continuum.^2^^,^^3^ Black/African American men have 70% more chances of being diagnosed with PCa and are over twice more likely to die because of this malignancy than their White/Caucasian American counterparts.^3^ Moreover, Black men tend to be diagnosed at a younger age than other races and respond poorly to the existing cancer therapies.^4^ Radical prostatectomy and radiation therapy are the most common choice of treatment for localized PCa patients, yet nearly 20%–40% PCa patients experience eventual disease relapse.^5^ Patients with locally advanced or metastatic disease receive androgen-deprivation or castration therapy as primary line of treatment but disease relapse is inevitable in nearly all patients sooner or later after an initial response.^5^^,^^6^

Prostate-specific antigen (PSA) is expressed by the cells of the prostate gland and its serum levels are often elevated in PCa patients.^7^ PSA is also an excellent biomarker to monitor disease recurrence following therapeutic intervention.^8^ Gleason grade of PCa is an established prognostic indicator and used in making treatment decisions in conjunction with clinical stage and serum PSA.^9^ PCa with a Gleason score of ≤6 is considered low grade and has a low risk of disease progression. Similarly, PCa with Gleason scores of 7 and ˃7 is considered medium and high grade, respectively, with an intermediate and high risk of faster tumor progression. PCa patients with high-grade tumors are more likely to have a metastatic disease than those with low-grade PCa and a higher chance of disease recurrence.^10^ There is, however, a significant heterogeneity in clinical outcomes within these risk groups, underscoring the need for improved risk stratification biomarkers. Interestingly, a recent study has shown that Black men with low-grade PCa are more likely to die from the disease than the men of other races^11^ highlighting the racial disparities and a need for additional markers for prognostication.

MYB, a proto-oncogene encoding a transcription factor, is involved in the regulation of several cancer-associated genes. Aberrant expression of MYB is reported in several liquid and solid malignancies, resulting from gene amplification or transcriptional or post-transcriptional upregulation.^12^^,^^13^^,^^14^^,^^15^ We have shown that MYB is aberrantly expressed in PCa exhibiting a higher expression in castration-resistant (CR) PCa cells, and promotes their growth, aggressiveness, and resistance to castration therapy.^16^ Androgen signaling regulates MYB expression in a biphasic manner, mediating its growth control in PCa.^17^ MYB interacts with androgen receptor (AR), facilitates its ligand-independent nuclear localization, and promotes its transcriptional activity.^18^ MYB likely also functions in an AR-independent manner since its overexpression is reported in AR non-expressing PCa cells as well.^16^

In this study, we have examined the expression of MYB in malignant prostate and adjacent benign prostatic hyperplasia (BPH) and high-grade prostate intraepithelial neoplastic (HGPIN) lesions from Black and White patients. Further, we have studied if MYB has an association with race, Gleason grade, pathological stage, AR expression, and time to biochemical recurrence (BCR). Our data reveal a racially disparate widespread expression of MYB in PCa. Aberrant MYB expression begins early in preneoplastic HGPIN, increases with advancing tumor grades and pathological stages, exhibits correlative expression with AR, and serves as a better predictor of BCR than nuclear AR and Gleason’s grades.

## Results

### MYB is overexpressed in prostate cancer and exhibits a positive association with increasing tumor grade

**Figure 1:**
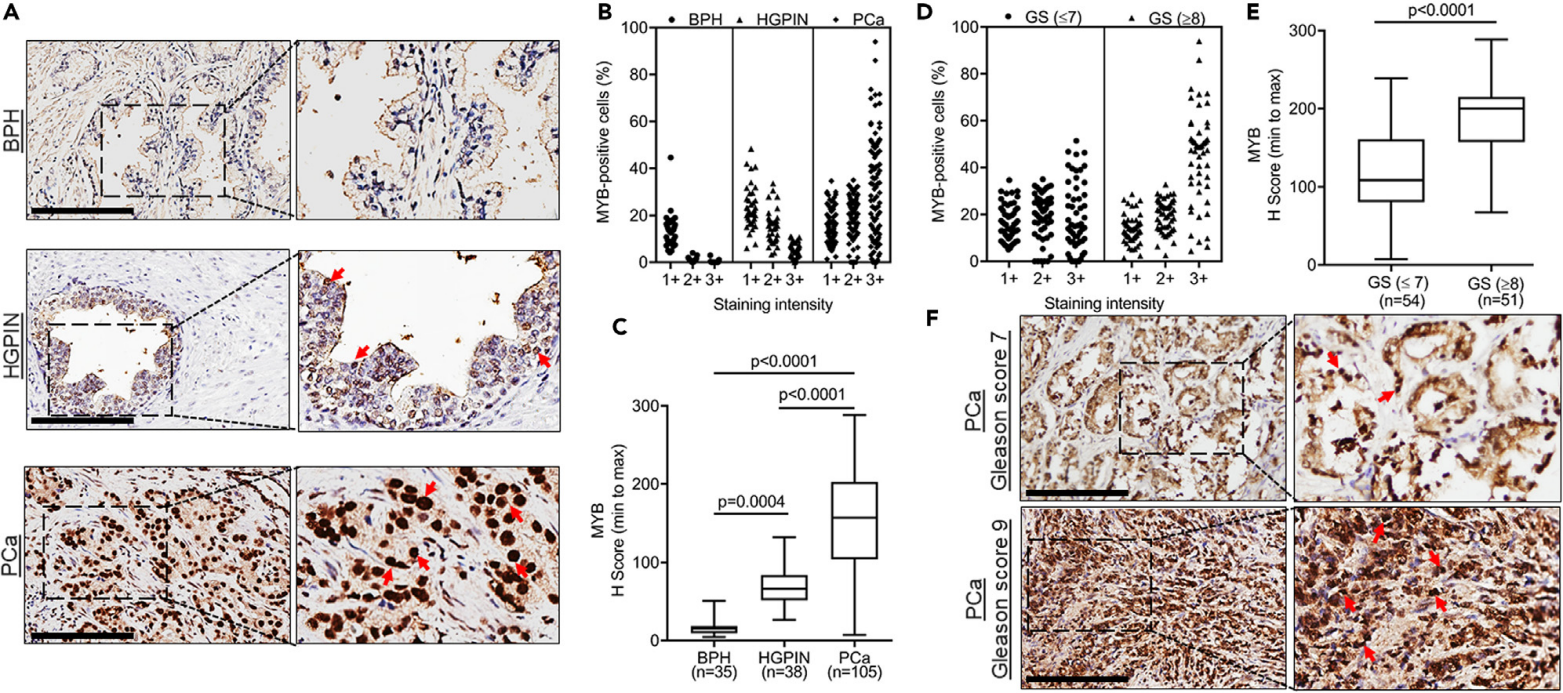
MYB is aberrantly expressed in prostate cancer and preneoplastic lesions and exhibits a positive association with advanced Gleason’s grades (A) MYB expression was analyzed by immunohistochemical staining in PCa (n = 105) along with adjacent BPH (n = 35) and HGPIN (n = 38) lesions. Representative images captured at 20X magnification (Scale bar: 200 μm; left panels) and digitally enhanced (2X) for improved visualization (right panels) are shown. Nuclear MYB staining is depicted by red arrows (B) Slides were scanned using Aperio ImageScope and digitally analyzed to assess percentage of positive cells with weak (1+), moderate (2+), and strong (3+) staining using Aperio’s Nuclear Image Analysis algorithm. Most cells in HGPIN exhibited weakly positive staining whereas weak-to-strong staining was observed in PCa. No or very weak staining was detected in BPH. (C) H score was calculated by multiplying staining intensity and percent positivity as described in STAR Methods section. The data are presented as a boxplot depicting median (horizontal line) value of each group. Top and bottom edges of the box represent 75th percentile (third quartile) and 25th percentile (first quartile), and whiskers point highest and lowest H scores. Kruskal-Wallis test was performed to compare the H-score between groups and a p value of <0.05 was considered statistically significant. (D and E) A higher percentage of cells in high Gleason grade (≥8) PCa (n = 51) stained strongly positive than low-medium grade (≤7) PCa (n = 54) resulting in significantly greater H score. Data are shown as a boxplot depicting highest and lowest MYB H score. Mann Whitney U test was used to compare the differences between low- and high-grade tumors and a p value of <0.05 was considered statistically significant. (F) Representative IHC images captured from low- (Gleason score 7) and high (Gleason score 9)-grade PCa (Scale bar: 200 μm; left panels) and digitally enhanced (2X) (right panels) are shown. Arrows depict nuclear MYB staining.

MYB expression was analyzed in prostate tumor tissues (n = 105), its adjacent BPH (n = 35), and HGPIN (n = 38) lesions (Table 1) by immunohistochemical analysis. Slides were scanned using an Aperio ScanScope and visualized on a computer monitor. MYB was found to be predominantly localized in the nucleus with some diffuse cytoplasmic expression (Figures 1A and S1). The extent of nuclear MYB staining was further analyzed using the Nuclear Image Analysis algorithm on the Aperio workstation, which provided the percentage of cells exhibiting weak (1+), moderate (2+), or strong (3+) staining (Figure S2). PCa cells exhibited weak to strong nuclear MYB staining, whereas mostly a weak staining was detected in HGPIN and negligible or a very weak expression of MYB was observed in BPH (Figure 1B). H score (percentage of positivity x staining intensity) comparisons showed statistically significant overexpression of MYB in PCa compared to HGPIN and BPH. MYB expression was also significantly higher in HGPIN than BPH lesions suggesting its deregulation begins at preneoplastic stages (Figure 1C). We next compared MYB expression between low-to-medium (n = 54; ≤7) and high (n = 51; ≥8) Gleason grade PCa. A higher percentage of cells in high Gleason grade PCa stained strongly positive for MYB compared to low-medium grade tumors contributing to overall significant MYB overexpression (Figures 1D–1F). Interestingly, comparison of MYB expression between low (n = 18; ≤6) and medium (n = 36; 7) Gleason’s grade PCa also showed a significant MYB overexpression in medium-grade PCa than low-grade PCa (Figures S3A and S3B). These data suggest that MYB may have a causal association with early and late events of prostate carcinogenesis.

**Table 1:** Details of prostate cancer patients used in the study

Information	Total	White	Black
PCa patients no.	105	50	55
Age mean ± S.D. (range)	59.5 ± 5.9 (41.4–72.2)	60.9 ± 4.9 (50.3–68.6)	58.2 ± 6.5 (41.4–72.2)
BPH^a^	35 (33.3%)	16 (15.2%)	19 (18.0%)
HGPIN^b^	38 (36.2%)	21 (20.0%)	17 (16.2%)
Gleason score (≤7)	54 (51.4%)	25 (50%)	29 (52.7%)
Gleason score (8–9)	51 (48.6%)	25 (50%)	26 (47.3%)
BCR^c^	41 (41.9%)	17 (34%)	24 (49%)

a **BPH:** Benign prostatic hyperplasia.

b **HGPIN:** High-grade prostatic intraepithelial neoplasia.

c **BCR:** Biochemical recurrence.

### MYB exhibits a racially disparate expression pattern in overall and grade-wise comparisons

**Figure 2:**
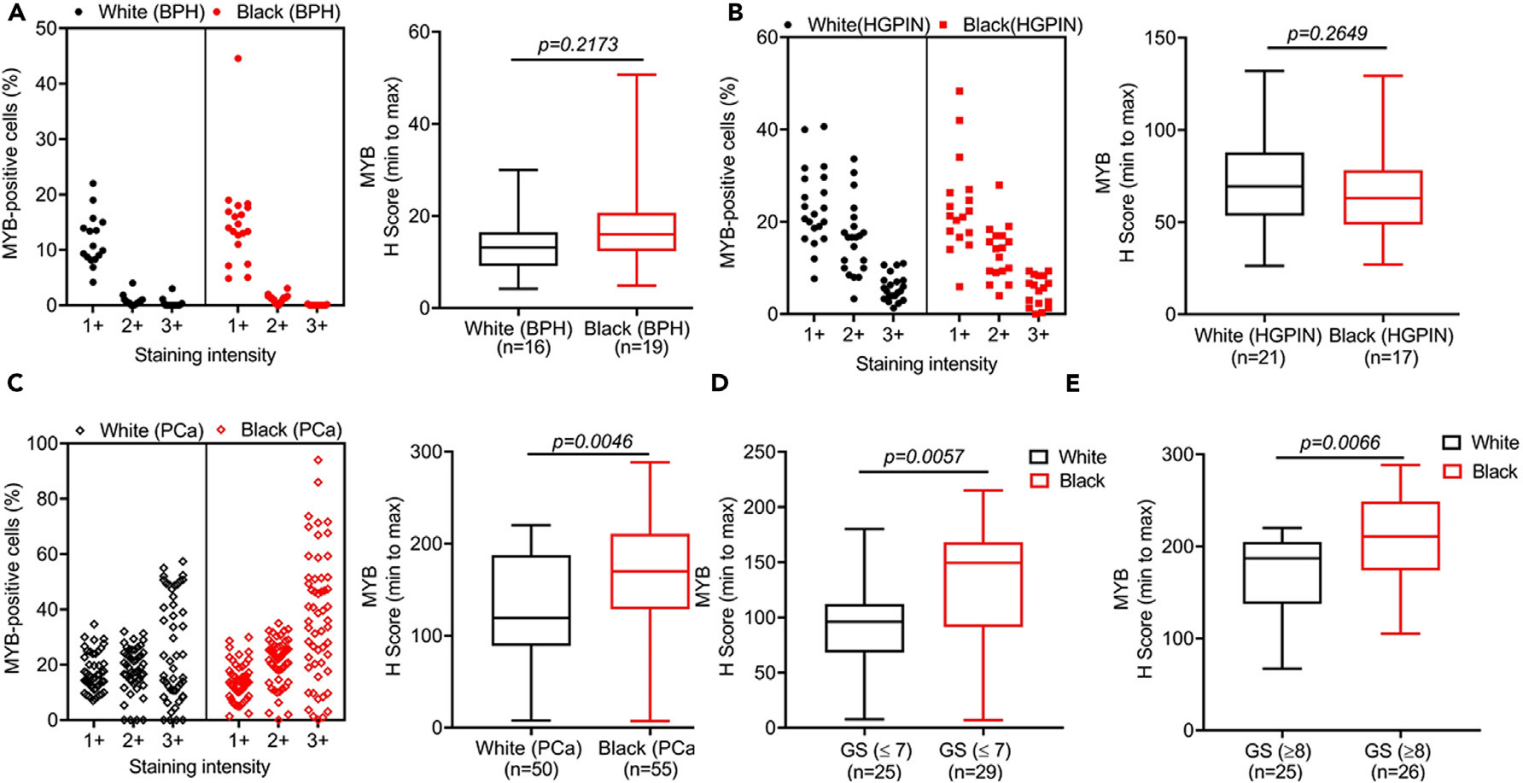
MYB expression is significantly higher in tumor tissues from Black patients than White patients (A) Due to a weak MYB staining in a fewer number of BPH cells, no significant differences were observed between White (n = 16) and Black (n = 19) patients. (B) Likewise, no significant difference in MYB positivity and H-scores were detected in the HGPIN lesions from White (n = 21) and Black (n = 17) patients. (C) MYB expression was significantly higher in the tumor tissues of Black patients (n = 55) than White patients (n = 50) with a greater percentage of cells exhibiting strongly positive staining in Black patients’ samples. (D and E) In a grade-wise comparison, an elevated MYB expression was observed in both low/moderate (≤7) and advanced grade samples of Black patients (≥8). The data are presented as a boxplot depicting median (horizontal line) value in each group. Top and bottom edges of the box represent 75th (third quartile) and 25th (first quartile) percentiles, respectively, and whiskers point highest and lowest H scores. The differences between groups were calculated by Mann Whitney U statistical test a p value of <0.05 was considered significant. GS: Gleason score.

Considering the prevalent racial disparity in the clinical outcomes between Black and White PCa patients, we analyzed if MYB expression in BPH, HGPIN, and PCa varied between these racial groups. No significant difference in MYB expression was observed in BPH and HGPIN lesions between White and Black patients; however, MYB expression was significantly higher (p = 0.0046) in the tumor tissues of Black patients as compared to White patients (Figures 2A–2C). Since we observed increased MYB expression in high-grade PCa compared to the low/medium-grade PCa, we also examined whether there was any racial association of MYB expression in grade-wise comparisons. Our analysis showed a significantly increased MYB expression in low/medium-grade (p = 0.0057) as well as high-grade (p = 0.0066) samples of Black patients than corresponding White patients (Figures 2D and 2E). Since Black PCa patients tend to have worse outcome than White PCa even with a low-grade tumor, these data suggest that MYB could be a better predictor of PCa aggressiveness than Gleason’s grades.

### AR is overexpressed in prostate cancer and exhibits race-specific differential expression in low-grade tumors

**Figure 3:**
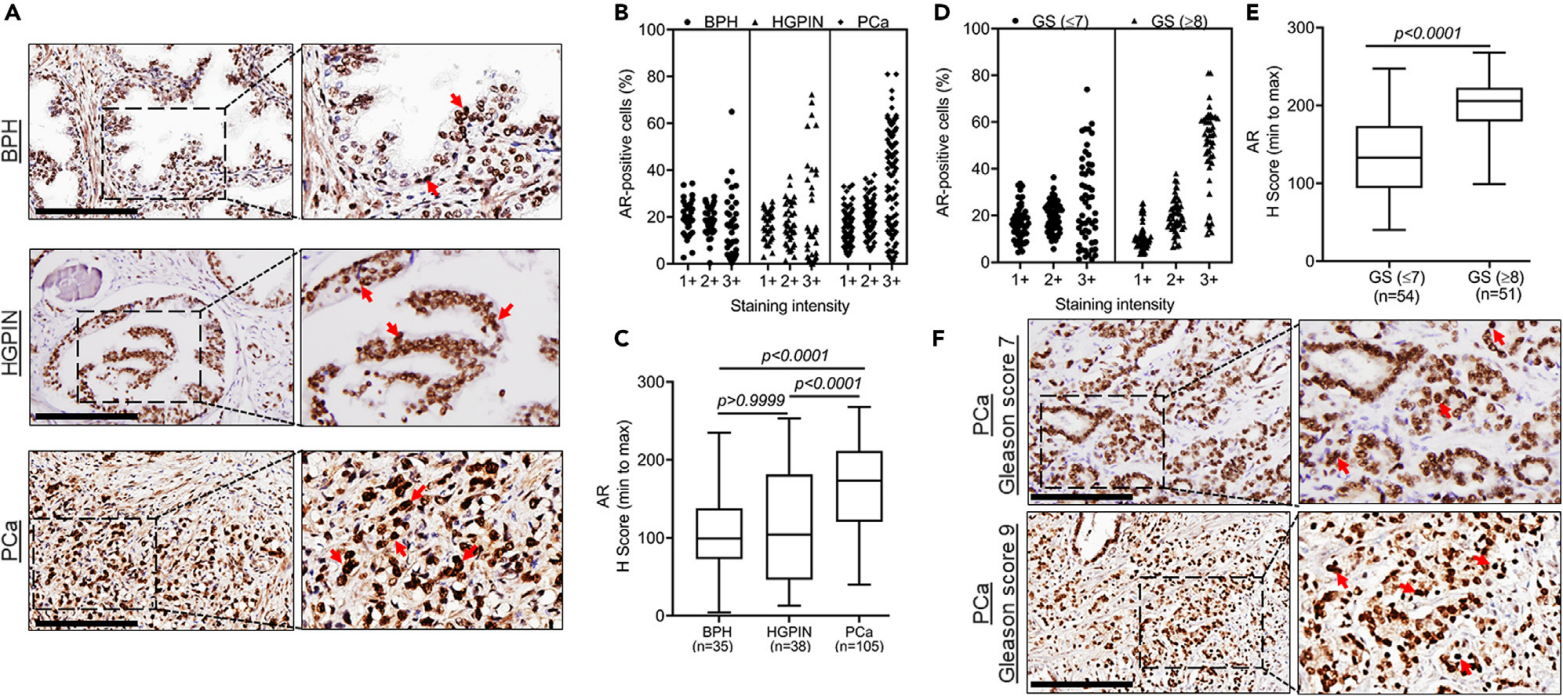
Androgen receptor expression in benign, high-grade prostatic intraepithelial neoplasia, and cancerous lesions of the prostate and its association with Gleason’s grade (A) AR expression was analyzed by immunohistochemistry in BPH (n = 35), HGPIN (n = 38), and PCa (n = 105). Representative images exhibiting nuclear AR staining (red arrow) were captured at 20X magnification (Scale bar: 200 μm; left panels) and digitally enhanced (2X) for improved visualization (right panels) are shown. (B) Digital analysis of scanned slides for nuclear AR expression by Aperio ImageScope shows a greater distribution of strongly positive cells in HGPIN and PCa lesions than in BPH. (C) AR expression is significantly higher in PCa as compared to the BPH and HGPIN in median H score comparison. No significant differences in AR expression between HGPIN and BPH are observed. The data are presented as boxplots showing highest and lowest H score. Kruskal-Wallis test was performed for comparing the differences between groups and a p value of <0.05 was considered statistically significant. (D and E) A higher percentage of cells in high Gleason (n = 51) grade PCa stained strongly positive compared to low-medium grade (n = 54) PCa leading to significant differences in overall AR expression. The data are presented as a boxplot depicting median (horizontal line) value of each group. Top and bottom edges of the box represent 75th percentile (third quartile) and 25th percentile (first quartile), and whiskers point highest and lowest H scores. The differences between the groups were calculated by Mann Whitney U test and a p value of <0.05 was considered significant. (F) Representative images showing a stronger nuclear AR immunostaining in high-grade (Gleason score 9) PCa relative to low-grade (Gleason score 7) PCa were captured at 20X magnification (Scale bar: 200 μm; left panels) and digitally enhanced (2X) for improved visualization (right panels) are presented. Nuclear AR staining is depicted by red arrows. GS: Gleason score.

**Figure 4:**
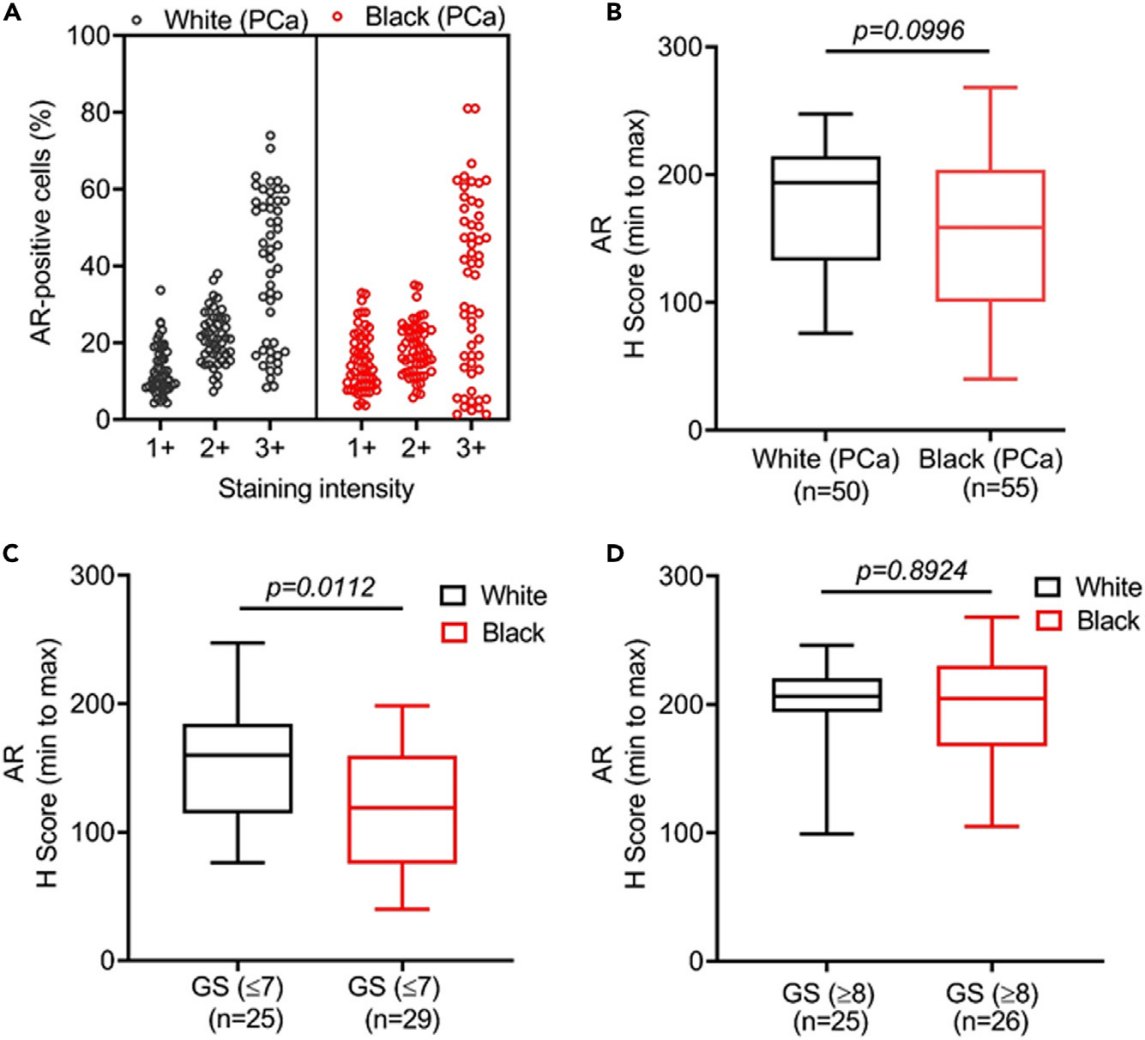
Androgen receptor expression in the tumor tissues of White and Black patients (A) The overall intensity pattern of AR positive cells was nearly same in the total cases of White (n = 50) and Black (n = 55) men. (B) The boxplot shows a reduction in the median AR H score value, although the difference between White and Black PCa patients is not statistically significant. (C and D) In a grade-wise comparison between White and Black PCa patients, significantly lower nuclear AR expression was observed in low-to-medium Gleason grade tumor samples of White (n = 25) than Black (n = 29); however, no significant differences were reported in high grade tumor tissues of White (n = 25) and Black (n = 26) patients. The data are presented as a boxplot depicting median (horizontal line) value of each group. Top and bottom edges of the box represent 75th percentile (third quartile) and 25th percentile (first quartile), and whiskers point highest and lowest H scores. Mann Whitney U test was performed to calculate the differences between groups and a p value of <0.05 was considered statistically significant. GS: Gleason score.

AR is a prime therapeutic target in PCa and androgen signaling has been implicated in PCa racial disparity.^6^^,^^19^ Therefore, we analyzed AR expression in BPH, HGPIN, and PCa from Black and White patients. A predominant nuclear expression of AR was detected with weak to moderate expression in BPH and HGPIN and a moderate to high expression in PCa (Figures 3A and S4). A higher percentage of PCa cells exhibited strong AR staining compared to HGPIN and BPH (Figure 3B). No significant differences in AR expression (p > 0.9999) were observed between BPH and HGPIN; however, its expression was significantly high in PCa than BPH and HGPIN (p < 0.0001) (Figure 3C). A significantly higher expression of AR (p < 0.0001) was detected in high-grade PCa than low-grade PCa because of higher percentage of cells exhibiting strong staining (Figures 3D–3F). In race-specific comparisons, we did not see any significant (p = 0.0996) difference in AR expression between White and Black PCa patients although a decreasing trend was observed in the tumor samples of Black patients (Figures 4A and 4B). In grade-wise comparison, a significantly reduced expression of AR (p = 0.0112) was reported in low-to-medium Gleason grade PCa in Black patients compared to White patients (Figure 4C). However, no significant (p = 0.8924) difference in AR expression was observed in high-grade PCa between White and Black patients (Figure 4D). These data suggest a complex relationship of AR signaling with racially disparate outcomes of PCa wherein other molecular targets may also be involved.

### MYB expression is positively correlated with AR in both Black and White prostate cancer patients

**Figure 5:**
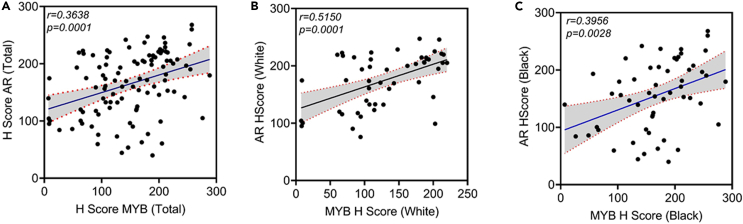
MYB shows a positive correlation with AR in prostate cancer cases (A) Pearson correlation coefficient (r) was calculated to examine the association between MYB and AR nuclear expression in PCa cases (n = 105). A significant positive association (r = 0.3638, p = 0.0001) was observed. Each point represents a single patient with its H score added on X (MYB) and Y (AR) axes. Regression line (solid) is drawn from a linear regression model between MYB and AR H scores with 95% confidence intervals (broken lines). (B and C) An improved positive correlation between MYB and AR is detected in White (n = 50; r = 0.5150, p = 0.0001) and Black (n = 55; r = 0.3956, p = 0.0028) PCa samples. A p value of <0.05 was considered as statistical significant.

Since we have observed an association of MYB and AR at the functional and regulatory levels, we examined if they both exhibit a correlative expression pattern in PCa. The data show a significantly positive correlation between MYB and AR (r = 0.3638, p = 0.0001) in PCa (Figure 5A). In race-wise comparisons, a stronger correlation of MYB and AR was detected in the PCa specimens from White patients (r = 0.5150, p = 0.0001) than Black patients (r = 0.3956, p = 0.0028) (Figures 5B and 5C). Interestingly, a weak, but positive, correlation between MYB and AR (r = 0.15, p = 0.00074) was also reported at the transcripts level in publicly available The Cancer Genome Atlas dataset (Figure S5). Since we observed MYB expression in HGPIN as well, we examined if its expression was correlated with AR in these lesions. A weak correlation between MYB and AR was observed in the overall as well as race-wise comparisons in HGPIN cases (Figures S6A–S6C).

### MYB exhibits a positive association with increasing pathological stages and is a better predictor of biochemical recurrence than AR in prostate cancer patients

**Figure 6:**
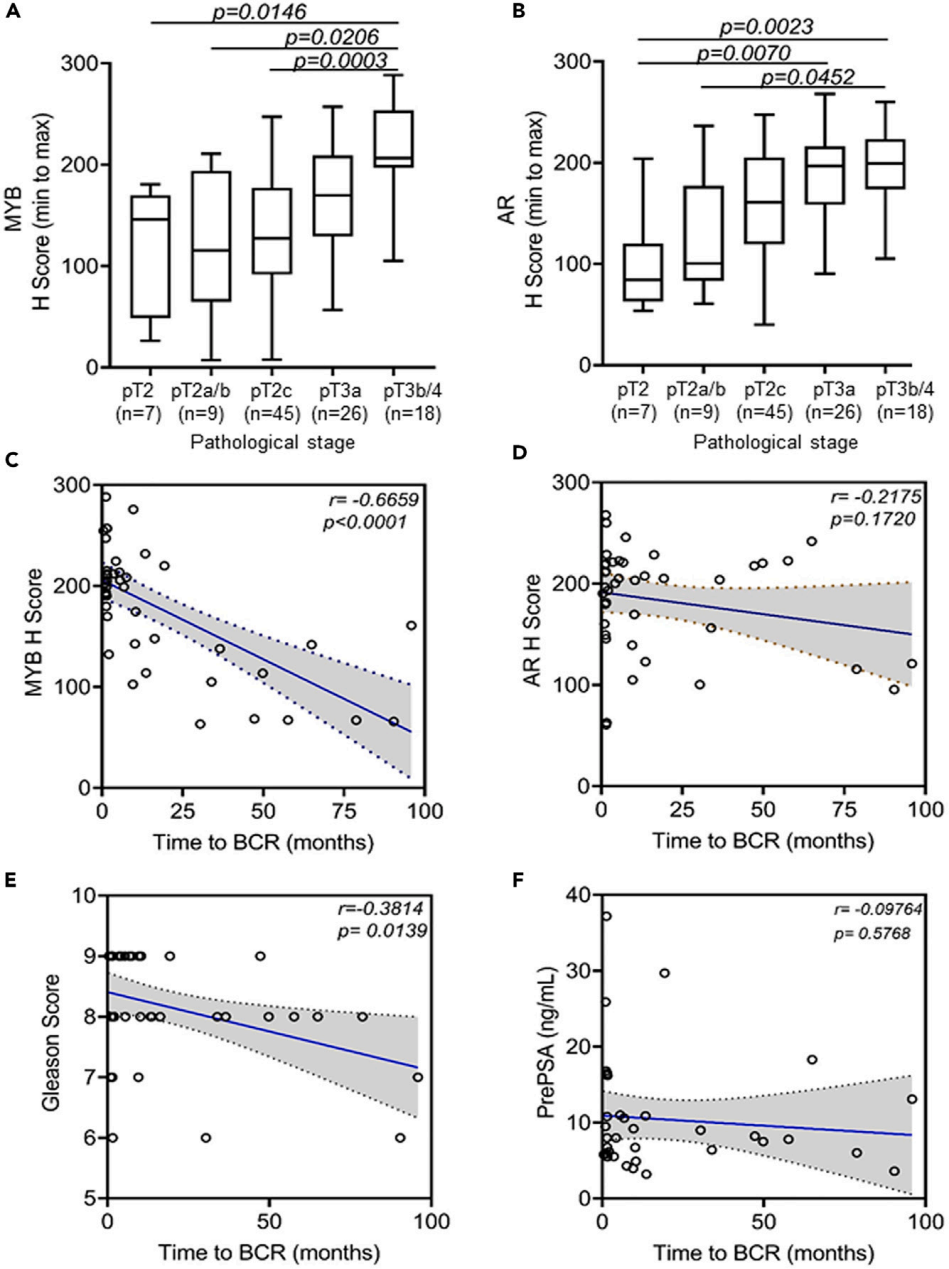
MYB and AR expression shows positive association with pathological stages, MYB and Gleason grades show negative association with biochemical recurrence in prostate cancer patients The boxplots of H scores depict the association of MYB (A) and AR (B) with different pathological stages (pT2-pT4) of PCa. The data are presented as a boxplot depicting median (horizontal line) value of each group. Top and bottom edges of the box represent 75th percentile (third quartile) and 25th percentile (first quartile), and whiskers point highest and lowest H scores. Expression of both MYB and AR increases as disease progress from pT2 to pT4. Kruskal-Wallis test was performed to determine the differences between groups and p value of <0.05 was considered as statistical significant. (C–F) Pearson’s correlation coefficient (r) was measured to establish the association of MYB, AR, Gleason’s grade, and PSA level at diagnosis (PrePSA) with time to biochemical recurrence (BCR) using cases with available information (n = 41). Both MYB and Gleason’s grade exhibit a significant negative correlation with time to BCR; however, MYB appears to be a better predictor (r = −0.6659; p < 0.0001) than Gleason’s grade (r = −0.3814; p = 0.0139). A negative but insignificant correlation of AR (r = −0.2175; p = 0.1720) and PrePSA (r = −0.09764; p = 5768) with time to BCR was observed. The regression (solid) line is shown with 95% confidence intervals (broken lines). A p value of <0.05 was considered significant.

We next analyzed the correlation of MYB and AR expression with progressive pathological stages (pT2-pT4) of the PCa in our cohort. A significantly higher expression of MYB and AR were observed in the tumor cells those were invaded into extraprostatic extension (pT3a), seminal vesicle (pT3b), or bladder and/or rectum (pT4) compared to prostate confined (pT2-pT2c) tumor (Figures 6A and 6B). A significant proportion of PCa patients exhibit BCR or PSA relapse following surgery and other optional treatments. Since both MYB and reactivation of AR signaling have been associated with PCa relapse, we examined if their expression would exhibit any correlation with the time to BCR in our cohort that had available information (n = 41). Furthermore, we also examined the correlation of time to BCR with the Gleason grading and PSA levels at the time of diagnosis (prePSA) as they are commonly used as prognostic indicators. We observed an inverse association of both MYB and AR with time to BCR; however, this association was remarkably stronger and significant for MYB (r = −0.6659, p < 0.0001) than AR (r = −0.2175, p = 0.1720) (Figures 6C and 6D). Furthermore, we found that MYB was a better predictor of BCR than Gleason’s grade, which also showed a negative and significant correlation with time to BCR (r = −0.3814, p = 0.0139) (Figure 6E). However, PrePSA showed a weak and insignificant association with time to BCR (r = −0.09764, p = 0.5768) (Figure 6F). Since, both MYB and AR cooperatively induce PSA expression, we examined if they had any correlation with pre-treatment PSA (Pre PSA). A weak but significant association of prePSA with MYB (r = 0.2502, p = 0.0174) but non-significant with AR (r = 0.1980, p = 0.0614) was observed (Figures S7A and S7B). Moreover, we observed a trend for increased prePSA levels in Black patients than White patients in overall and grade-wise comparisons (Figures S8A–S8C) but could not see any trend between the level of prePSA and pathological stage (Figure S8D).

## Discussion

PCa management is challenging due to its highly heterogeneous nature.^20^^,^^21^ Most patients are diagnosed early when the cancer is still confined to the prostate that can be removed by radical prostatectomy and/or subjected to radiation therapy. If the disease is locally advanced or has metastasized, patients are subjected to hormone therapy or androgen deprivation therapy. The disease relapse is, however, common and it cannot be precisely predicted using currently available parameters, i.e., Gleason’s grade, serum PSA levels, and the clinical stage of the disease.^5^^,^^10^^,^^22^ PSA is often criticized for PCa overdiagnosis leading to unnecessary treatment of patients that might have a slow progressing disease.^23^ Similarly, race-specific differential outcomes have been reported for patients with low-grade PCa.^11^ In these contexts, our findings are highly promising and hold potential to improve the management of PCa.

Molecular biomarkers with established functions in disease pathobiology can be superior to histology-based stratification, especially in cancers that have high genetic heterogeneity. They can be more reliable and used alone or in combination with other stratification criteria to diagnose, predict patients’ prognosis, and make better treatment decisions. Extensive use of genomics and transcriptomics have aided in the identification of novel molecular targets exhibiting genetic alterations and differential abundance in tumors.^24^^,^^25^ These targets have further been validated for their functional involvement in disease processes and therapeutic outcomes. MYB was reported to be amplified in PCa about two decades ago and exhibited increased amplification frequency in CR PCa.^26^ Subsequently, it was shown to promote PCa growth, aggressiveness, and castration resistance.^16^ Current findings provide additional clinical support for these experimental and preclinical observations and also suggest an association of MYB with race-associated disparate health outcomes.

Androgen signaling is central to PCa growth and progression. As a result, rise in serum PSA, a protein encoded by an AR target gene, KLK3, is used in PCa screening and monitoring following therapeutic intervention.^6^^,^^27^ A subgroup of PCa, especially CR PCa, lack AR expression, while in others AR signaling remains active following androgen suppression via a variety of mechanisms.^6^^,^^28^ An important role of AR signaling is also suggested in PCa racial disparities; however, there are discrepancies in related observations.^29^^,^^30^ In our cohort, we observed greater nuclear AR expression in PCa than BPH and HGPIN, which also exhibited positive association with advanced Gleason grades. There were, however, no significant difference between White and Black patients in overall comparisons and a significantly reduced expression of AR was observed in low-grade PCa samples. This is interesting and should be investigated further in light of the fact that an aggressive subgroup of PCa, referred as neuroendocrine type, is characterized by a loss of AR.^31^

This study showed significant MYB overexpression in PCa, which correlated with race, grade, and pathological stage of the patients. Aberrant MYB expression was also noticeable in HGPIN suggesting that its dysregulation starts early and may be involved in initial stages of prostate carcinogenesis. Further, we observed a highly prevalent MYB overexpression although at varying levels in PCa suggesting that both genetic (gene amplification) and transcriptional and post-transcriptional mechanisms may be involved. Indeed, we have observed that androgen signaling may have a biphasic effect on MYB expression involving its transcriptional and post-transcriptional regulation.^17^ A positive association of MYB with increasing tumor grade and stage may result from its involvement in epithelial-mesenchymal transition and gain of aggressive phenotypic traits.^16^ Along with these observations, relative MYB overexpression in the PCa from Black patients is also of significant interest considering their aggressive nature and poor response to therapeutic interventions.^2^^,^^19^^,^^32^ Therefore, it is imperative to delineate the mechanisms involved in higher MYB levels in PCa from Black patients. It is likely that certain race-specific genetic/epigenetic alterations are involved in the differential expression of MYB in Black vs. White patients, which should be explored in future studies.

Earlier, we found that MYB interacts with AR and modulates its transcriptional activity.^18^ Further, we observed that androgens regulated MYB expression in a biphasic manner and MYB induction was essential for growth-promoting action of androgen.^17^ In this study, we observed that MYB expression correlated with AR nuclear positivity in both Black and White patients’ samples. This is interesting and suggests that cooperative MYB-AR functions might play a significant role in PCa pathobiology. Nonetheless, it will also be interesting to investigate AR-independent functions of MYB since we observed its overexpression in AR non-expressing in PCa cell lines as well.^16^ Moreover, MYB and AR exhibit opposite expression pattern in low-grade PCa between Black and White patients’ samples. Thus, MYB-AR relationship appears complex and they both may work independently or through other interacting partners to have a diverse functional impact on PCa pathobiology.

Rise in PSA levels following early local therapeutic interventions signals the PCa relapse or BCR likely resulting from the undetected metastatic lesions at the time of diagnosis or positive surgical margins in radical prostatectomy.^33^ Gleason is grading along with clinical stage and serum PSA levels are used as predictors of therapeutic outcomes and stratifying the patients into different risk groups.^22^^,^^34^ However, this system is not efficient in risk prediction and finding additional biomarkers for indolent versus aggressive disease remains a prime are of PCa research. Our study shows that MYB is a better predictor of time to BCR than Gleason grades. Nuclear AR also showed positive predictive value for early BCR but it was poorer than Gleason’s grades. This is an exciting observation and should be explored in larger cohorts in combination with other parameters, including Gleason grade, prePSA level, and clinical stage for improved risk prediction. Indeed, 3 out of 4 low-grade PCa that had an early BCR exhibited a relatively higher MYB (H score: ≥150) and were from Black patients.

Altogether, our study establishes MYB as a molecular target of high significance for improved clinical management alone and in combination with other existing parameters. Since MYB dysregulation starts early and increases progressively, it is liked involved in early and late events of prostate carcinogenesis. Further investigations may thus establish MYB as an attractive target for both cancer prevention and therapy. In addition, racially disparate expression of MYB in Black and White prostate cancer warrants future studies to characterize mechanisms underlying its dysregulation in these racial subgroups and investigate if MYB targeting could be helpful in closing the disparity gaps.

### Limitations of the study

One potential limitation of our study is that all racial designations are based on self-reporting. Race is a social construct that cannot be defined based on physical and genetic parameters. However, in future studies, we can perform ancestry profiling to see if the people of a higher percentage of African ancestry tend to have a higher expression of MYB or if the MYB expression is somehow associated with race-associated social experiences affecting tumor biology via epigenetic mechanisms. Another limitation is that our association of MYB with time to biochemical recurrence is based on a smaller sample size that prevented us from race-based comparisons. Lastly, we lacked prostate cancer cases from metastatic lesions and relapsed disease to derive additional clinical support. Thus, a multi-center study in larger cohorts of samples would be needed to validate our findings and bringing them closer to clinical practice.

## STAR★Methods

### Key resources table

**Table:** 

REAGENT or RESOURCE	SOURCE	IDENTIFIER
**Antibodies**		

Rabbit polyclonal anti-*c*-Myb	Abcam	Cat# ab226470; RRID: AB_3075499
Rabbit monoclonal anti-AR	Cell Signaling	Cat# 5153S; RRID: AB_10691711

**Biological samples**		

Human prostate tumor tissues	Eastern Virginia Medical School (Norfolk, VA, USA)	N/A

**Chemicals, peptides, and recombinant proteins**		

Peroxidazed 1	Biocare Medical	Cat# PX968MM
Da Vinci Green Diluent	Biocare Medical	Cat# PD900H
Betazoid DAB Chromogen Kit	Biocare Medical	Cat# BDB2004L
MACH 3 Rabbit HRP Polymer Detection	Biocare Medical	Cat# M3R531H
Nuclear Decloaker, 10X	Biocare Medical	Cat# CB911M
TBS Automation Wash Buffer, 20X	Biocare Medical	Cat# TWB945M
Background Sniper	Biocare Medical	Cat# BS966L
Tacha’s Bluing Solution	Biocare Medical	Cat# HTBLU-M
Xylene	Thermo Fisher Scientific	Cat# 447240010
Ethanol	Fisher Scientific	Cat# A405P-4
Hematoxylin	Vector Laboratories	Cat# H-3401-500
VectaMount Permanent Mounting Medium	Vector Laboratories	Cat# H-5000

**Deposited data**		

Mendeley dataset	Mendeley	Mendeley Data: https://data.mendeley.com/preview/w5xvb9xr8s?a=22cafa50-6520-4c5b-a19d-2492793b77cd

**Software and algorithms**		

GEPIA2	GEPIA2	GEPIA2: http://gepia2.cancer-pku.cn/#correlation
GraphPad Prism 8.0	GraphPad	GraphPad: https://www.graphpad.com/
Adobe Photoshop	Adobe Inc.	Adobe Photoshop: https://www.adobe.com/products/photoshop.html
Code		This paper does not report the original code.

### Resource availability

#### Lead contact

Requests for additional information regarding the resources and reagents should be directed to the lead contact, Ajay Pratap Singh (asingh@southalabama.edu).

#### Materials availability

Our study did not generate any unique reagents and all the reagents used in this study are commercially available.

#### Data and code availability

• The RNA data analyzed using the web analytical platform (GEPIA2: http://gepia2.cancer-pku.cn/#correlation), was from The Cancer Genomic Atlas (TCGA) database.
• This paper does not report the original code.
• Any additional information required to reanalyze the data reported in this paper is available from the lead contact upon request.

### Experimental model and study participant details

#### Tissue samples, antibodies, and reagents

Archival de-identified, formalin-fixed paraffin-embedded (FFPE) prostate tumor tissue sections were obtained from the Eastern Virginia Medical School (Norfolk, VA, USA) under an exempt protocol as determined by the Institutional Review Board (IRB) of the University of South Alabama (Table 1). Anti-MYB (cat #ab226470) and anti-AR (cat #5153S) rabbit antibodies were obtained from Abcam (Cambridge, MA) and Cell signaling (Danvers, MA), respectively. Peroxidazed 1, Da Vinci Green diluent, betazoid DAB Chromogen Kit, MACH 3 Rabbit HRP Polymer Detection, Nuclear Decloaker, wash buffer (Tris-buffered saline with Tween 20), Background Sniper, and Tacha’s bluing solution procured from Biocare Medical (Concord, CA). Xylene and histological grade ethanol was purchased from Thermo Fisher Scientific (Waltham, MA).

### Method details

#### Immunohistochemistry

Immunohistochemistry was performed on FFPE prostate tumor tissue sections and adjacent BPH and HGPIN. Briefly, tissue sections were deparaffinized, hydrated, and processed to heat induced antigen retrieval in Decloaking Chamber (Biocare Medical). Afterward, endogenous peroxidase activity was blocked by incubating the slides in 30% H_2_O_2_ in methanol at room temperature for 30 min followed by background blocking for 10 min with Background Sniper. Next, slides were incubated with anti-MYB or anti-AR antibody overnight at 4°C. Subsequently, slides were washed with Tris-containing buffer and incubated with respective polymer and probe for 10 min each at room temperature. Immunoreactivity was visualized by incubating the sections with diaminobenzidine (DAB) chromogen followed by hematoxylin counterstaining.

#### Digital image analysis

Following IHC staining, slides were mounted with VectaMount Permanent Mounting Medium (Vector Laboratories, Newark, CA) and scanned at 20x magnification on Aperio CS2 whole slide scanner (Leica Biosystems, Deer Park, IL). High-quality digital images were used for measuring the staining intensity and percent positivity for nuclear MYB and AR expression using Aperio algorithm on Image Analysis workstation. Cells were given a score of +1, +2, or +3 for low, medium, and high staining intensity, respectively. A cumulative H score was calculated by multiplying this value with percent positive cells for each staining intensity [{1x (% positive cells with 1+staining)} + (2x (% positive cells with 2+ staining)} + (3x (% positive cells with 3+ staining)}]. Possible range for H score was 0–300 for no staining to 100% of cells exhibiting strong reactivity.

#### *In silico* data analysis

Publicly available TCGA database was interrogated using GEPIA [(Gene Expression Profiling Interactive Analysis) platform (GEPIA2: http://gepia2.cancer-pku.cn/#correlation)] to examine a correlation expression of MYB and AR at the transcript level.

### Quantification and statistical analyses

Statistical analyses were carried out using GraphPad Prism 8 software (GraphPad Software, San Diego, CA). Mann Whitney U or Kruskal-Wallis tests were used for comparing two or multiple groups, respectively. For the correlation studies, Pearson correlation coefficients with their significance were calculated and presented as scattered plots. Slope was generated by simple linear regression curve-fit analysis. P-values were two-sided with confidence intervals of 95%. A p values of less than 0.05 was considered statistically significant.
