# Supplementary material for: MYB exhibits racially disparate expression, clinicopathologic association, and predictive potential for biochemical recurrence in prostate cancer
Source: iScience. 2023 Nov 19;26(12):108487. doi: 10.1016/j.isci.2023.108487 (PMC10711386; doi:10.1016/j.isci.2023.108487)
Supplement: Document S1. Figures S1–S8 [file mmc1.pdf]

iScience, Volume *26*

Supplemental information

MYB exhibits racially disparate expression, clinicopathologic association, and predictive potential for biochemical recurrence in prostate cancer

Mohammad Aslam Khan, Srijan Acharya, Shashi Anand, Fnu Sameeta, Paramahansa Pramanik, Christopher Keel, Seema Singh, James Elliot Carter, Santanu Dasgupta, and Ajay Pratap Singh

Supplementary Figures and Legends

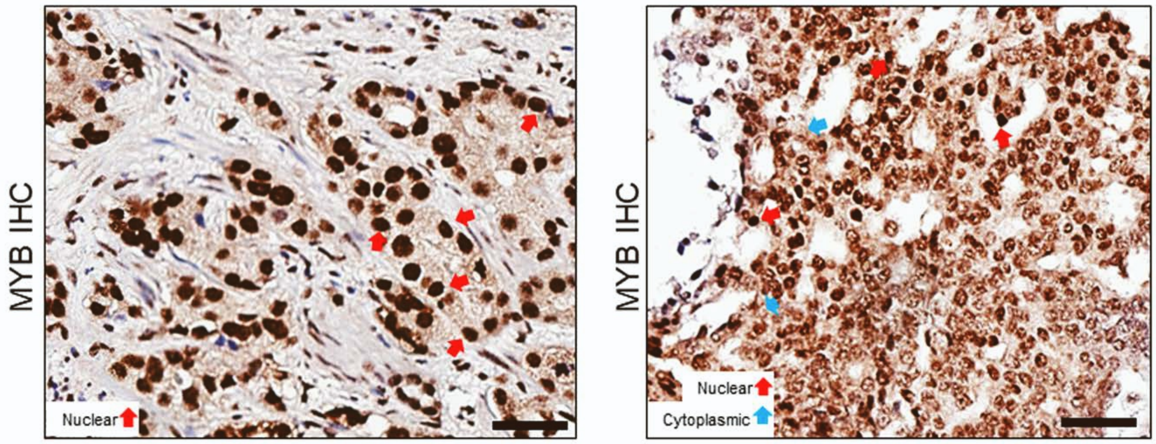

Figure S1. Immunohistochemical staining pattern of MYB in prostate tumor tissues. Prostate tumor tissue were stained by immunohistochemistry using MYB antibody. Slides were scanned at 20x magnification using the Aperio whole slide scanner and enhanced (2X) digitally (Scale bar, 50µm). The representative images are shown exhibiting predominantly nuclear MYB staining (red arrows) with some diffuse cytoplasmic staining (blue arrows) in some PCa cases.

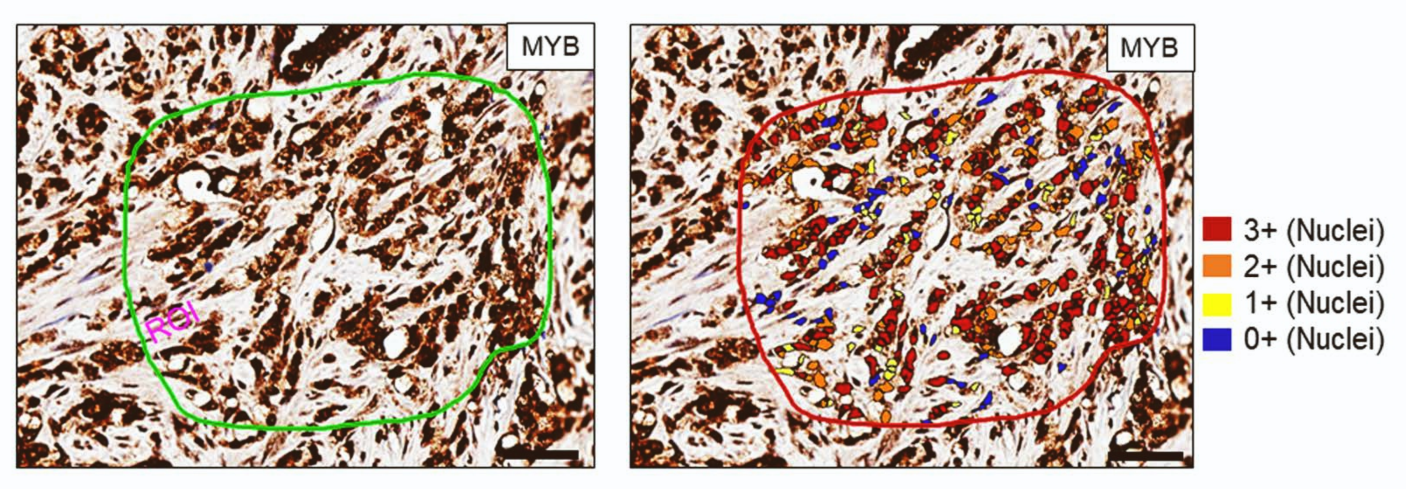

Figure S2. Digital image analysis of MYB expression in prostate tumor tissue. Aperio Image Analysis algorithm was used to analyze MYB (brown staining) in PCa tissue sections. Scanned slides were open in eSlide Manager to visualize and select the regions of interest (ROI) (green borderline). Aperio Image Analysis algorithm detected nuclear stain of antigen (MYB) chromogen in the tumor cell nuclei and quantified the antigen (MYB) staining intensity. The staining intensities were assigned into a color coded pattern where blue indicated negative (0) staining and yellow, orange, and red indicated weak (1)+, moderate (2+), and strong (3+) strong staining respectively. Scale bar, 50μm.

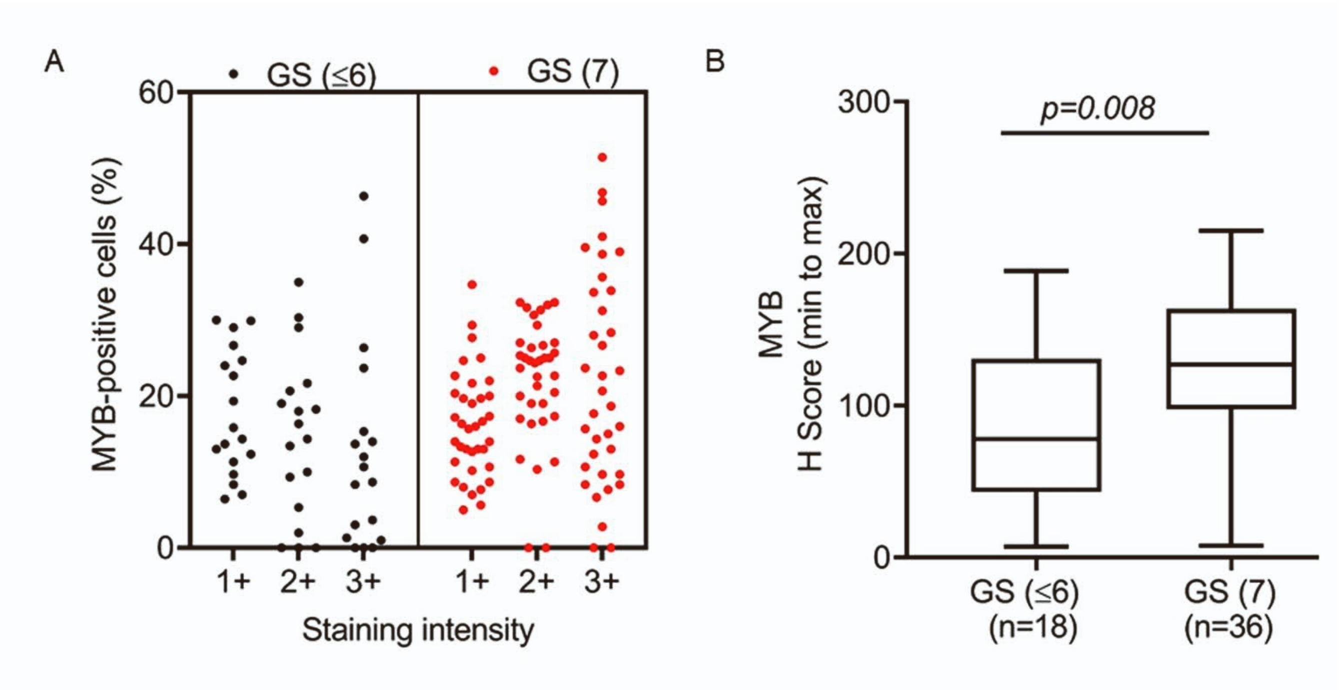

Figure S3. Comparison of MYB protein expression in low (≥6) and medium Gleason (7) grade samples of prostate cancer patients. (**A**) The percentage of tumor cells in medium grade tumors (n=36) show a higher percentage of strong MYB staining than that low grade (n=18) tumors. (**B**) Consequently, a higher median value of H score is reported in the medium Gleason grade tumor tissues than low grade tumors, and the difference is statistically significant. The data is presented as a box plot depicting median (horizontal line) value of each group. Top and bottom edges of the box represent 75th percentile (third quartile) and 25th percentile (first quartile), and whiskers point highest and lowest H scores. The difference between groups calculated by Mann Whitney U test and a p-value < 0.05 was considered statistically significant. GS: Gleason score.

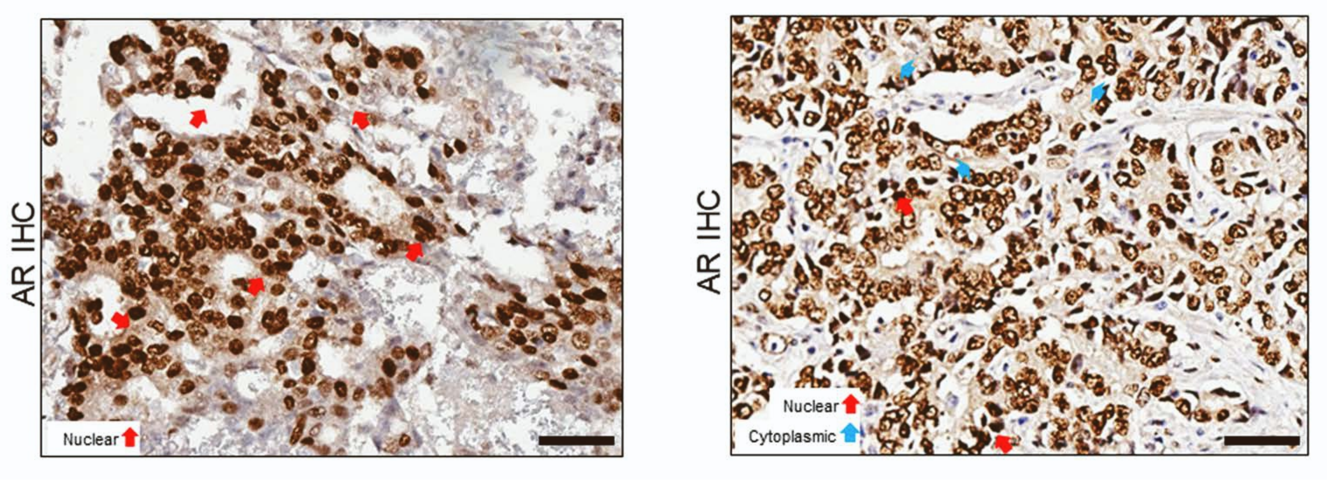

Figure S4. AR staining pattern in prostate tumor tissues. Prostate tumor specimens were stained by immunohistochemistry using AR-specific antibody. Slides were scanned at 20x magnification using the Aperio whole slide scanner. The representative IHC images are shown. Nuclear and cytoplasmic AR staining is depicted by red and blue arrows, respectively. Scale bar, 50μm.

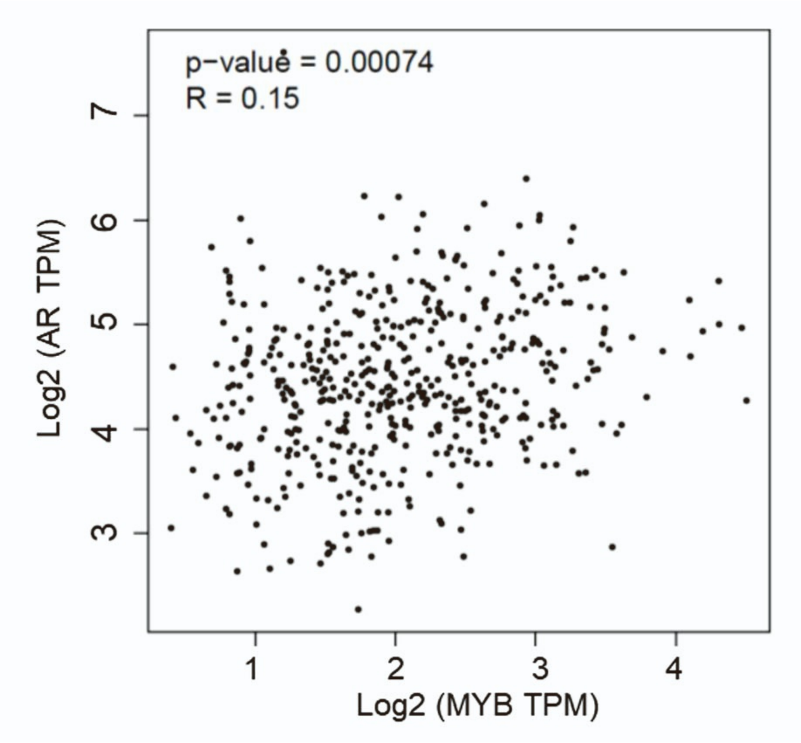

Figure S5. MYB and AR transcripts show a positive association. A weak positive association between MYB and AR transcript levels was also detected by performing Pearson correlation coefficient analysis of available TCGA data on Gene Expression Profiling Interactive Analysis2 (GEPIA2) platform ($r=0.15$, $p=0.00074$). A p value < 0.05 was considered statistically significant.

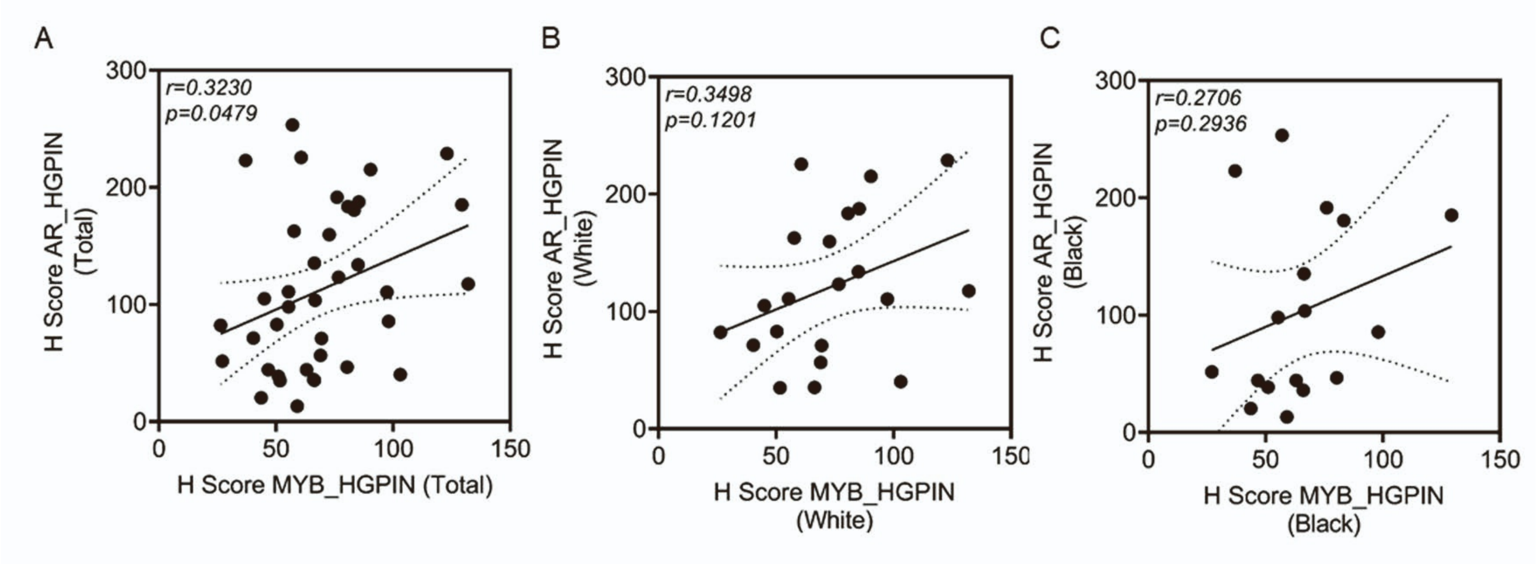

Figure S6. MYB shows a weak positive correlation with AR in HGPIN samples. (**A**) Pearson correlation coefficient (r) determines the association between MYB and AR expression in HGPIN (n=38) samples. A weak positive association (r=0.3230, p=0.0479) between MYB and AR was observed in the total HGPIN cases. (**B** and **C**) Furthermore, the correlation between MYB and AR in White HGPIN (n=21; r=0.3498, p=0.1201) and Black HGPIN (n=17; r=0.2706, p=0.2936) samples were also weak and non-significant. A statistically significant p-value ($p < 0.05$) was used.

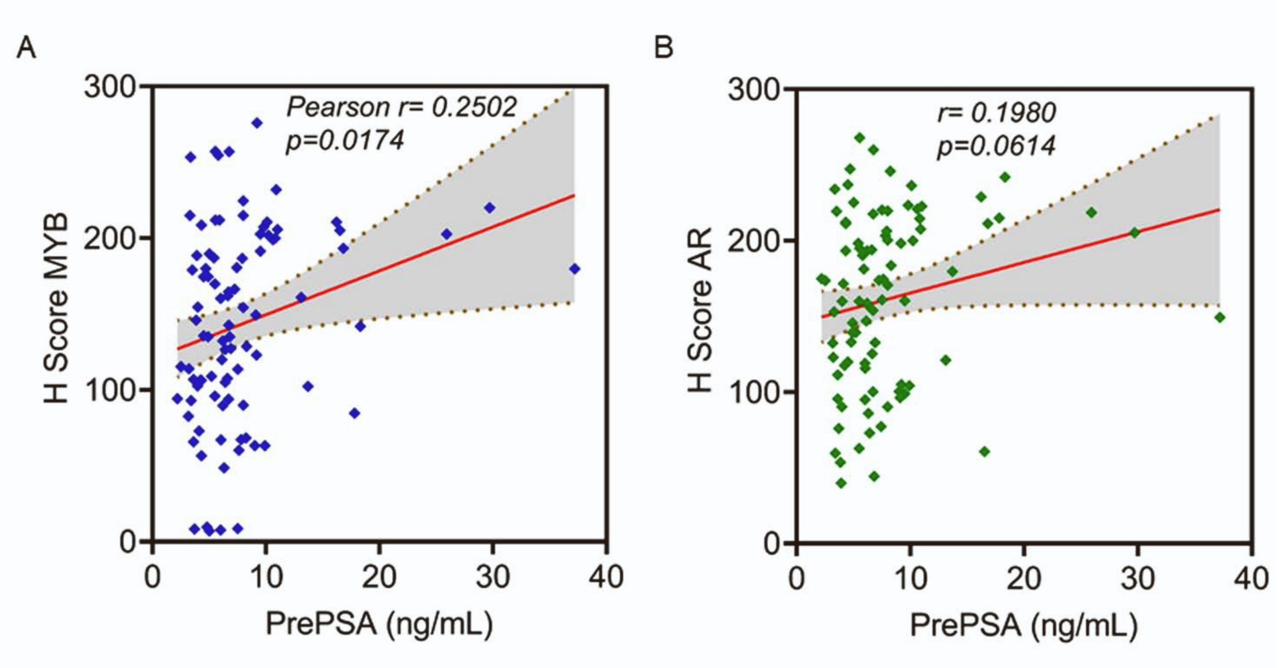

Figure S7. Expression of MYB and AR shows a poor correlation with prePSA. Since both MYB and AR regulate PSA expression, Pearson correlation coefficient (r) was estimated to examine their correlation [MYB (**A**) and AR (**B**)] with PrePSA (PSA levels that were recorded at the time of diagnosis or prior to the surgery). A weakly positive association of MYB (r=0.2502, p=0.0174) and AR (r=0.1980, p=0.0614) was detected with prePSA suggesting the extent of tumor as a major confounding factor.

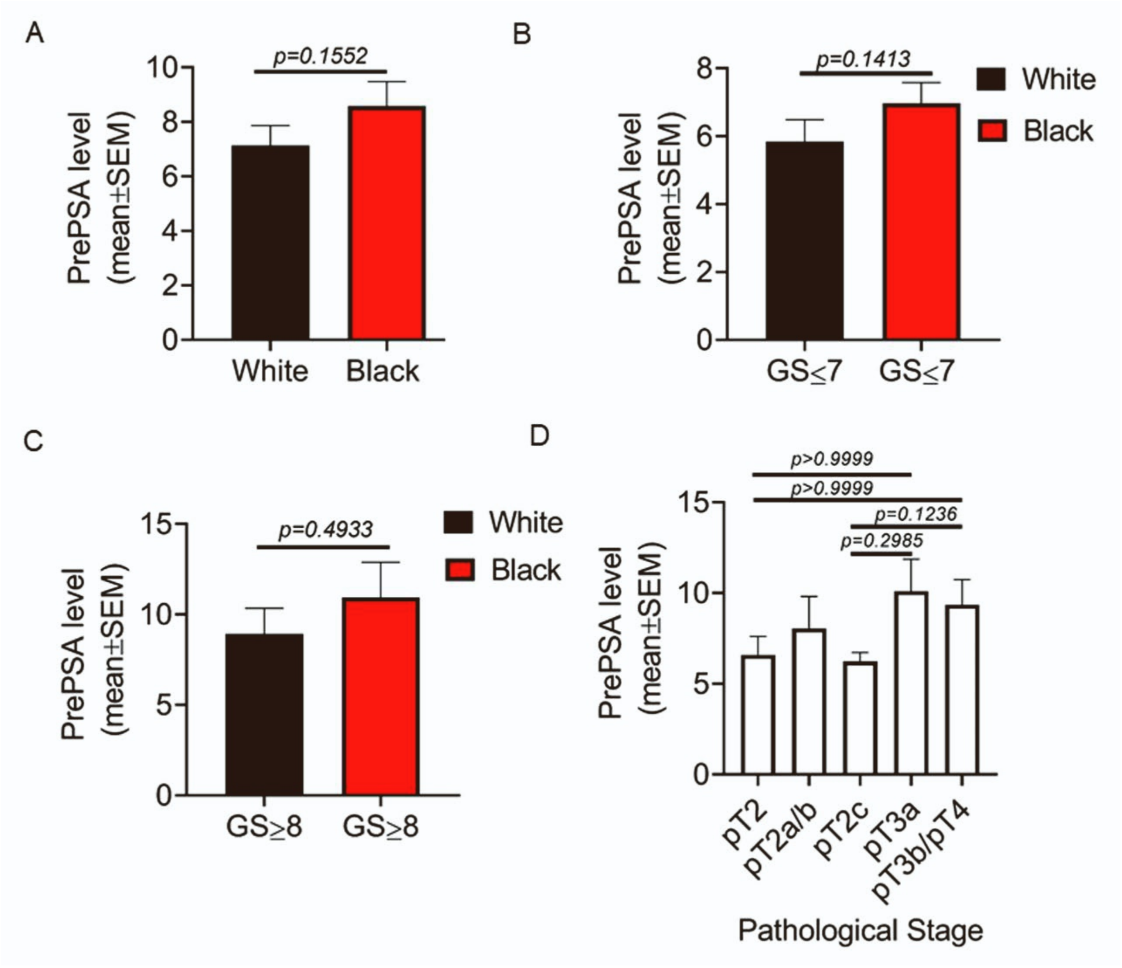

Figure S8. PrePSA level in White and Black prostate cancer patients. The mean PrePSA level was higher in the samples of Black (n=47) PCa patients than White (n=43) patients (**A-C**) and in grade-wise comparison, increased level of PrePSA was observed in low/moderate (≤7) or advanced grade (≥8) samples of Black PCa patients compared to White patients. Data are represented as mean +/- SEM. Mann Whitney U test was used to calculate the differences between groups and a p value < 0.05 was considered statistically significant (**D**) No trend was seen in the stage-wise comparison between PrePSA and pathological stage. Data are represented as mean +/- SEM. Kruskal-Wallis statistical test was performed and a p value of < 0.05 was considered significant. GS: Gleason score.
